# Assessing the Effectiveness of Crowdsourced Data to Detect Established Tick Populations in Quebec, Canada: Retrospective Ecological Study

**DOI:** 10.2196/90734

**Published:** 2026-08-19

**Authors:** Ariane Dumas, Jade Savage, Kirsten E Crandall, Jules K Koffi, Patrick A Leighton, Nicholas H Ogden, Erin E Rees, Catherine Bouchard

**Affiliations:** 1 Modelling Hub Division, Applied Public Health Sciences Directorate, Science and Policy Integration Branch, Public Health Agency of Canada, 200, boulevard René-Lévesque Ouest, Tour Est, Montréal, QC, H2Z 1X4, Canada, 1 514-498-5522; 2 Groupe de Recherche en Épidémiologie des Zoonoses et Santé Publique, St-Hyacinthe, QC, Canada; 3 Département de pathologie et microbiologie, Faculté de médecine vétérinaire, Université de Montréal, St-Hyacinthe, QC, Canada; 4 Department of Biology and Biochemistry, Bishop’s University, Sherbrooke, QC, Canada; 5 Unité Maladies infectieuses en communauté, Direction des risques biologiques, Institut national de santé publique du Québec, Montreal, QC, Canada; 6 Zoonoses Division, Centre for Foodborne, Environmental and Zoonotic Infectious Diseases, Public Health Agency of Canada, Saint-Hyacinthe, QC, Canada

**Keywords:** surveillance, tick-borne disease, Lyme disease, crowdsourced data, tick exposure, tick digital images, participatory science, socioecological factors, climate suitability, landcover

## Abstract

**Background:**

The range expansion of ticks transmitting zoonotic diseases in Canada poses challenges for public health surveillance. Active monitoring of where tick populations are emerging helps inform public health responses, but is resource intensive and logistically complex. Crowdsourced data may provide a cost-effective approach to augment surveillance. In this study, we assessed the value of crowdsourced data from eTick, a platform whereby the public submits tick photographs for expert identification.

**Objective:**

We aimed to (1) characterize spatial patterns of eTick submissions and identify socioecological factors associated with their occurrence and frequency, and (2) assess the ability of indicators derived from eTick to predict census subdivisions (CSDs) with established tick populations.

**Methods:**

We conducted a retrospective ecological assessment (2020‐2023) for the effectiveness of crowdsourced data to detect established blacklegged tick (*Ixodes scapularis*) populations in Quebec, Canada. First, we applied a spatial scan approach to assess significantly high submission rates in human- and animal-origin data separately. Then, using zero-inflated regression models, we assessed socioecological factors associated with the occurrence and counts of tick submissions. Next, we used logistic regression models to assess the value of using eTick surveillance data to predict the presence of established tick populations, as documented by active surveillance. Finally, we calculated thresholds for eTick submissions to maximize sensitivity, specificity, or a balance of both metrics for detecting established tick populations, and developed scenarios to interpret linear predictors of submission counts across different socioecological contexts.

**Results:**

No clusters in submissions were detected for human and animal-origin data. However, high concentrations of ticks found on humans corresponded with areas reporting a high risk of Lyme disease. Tick submissions of animal origin extended beyond endemic areas, highlighting their complementary surveillance value. Zero-inflated models identified socioeconomic, climatic, and land cover factors significantly associated with submission rates. Logistic regression models, including significant socioecological factors, performed best to identify CSDs with established tick populations. Predictive performance was similar for ticks from humans (area under the curve [AUC] 0.86), animals (AUC 0.83), and all submissions combined (AUC 0.85). Using the best model adjusted for population size, median income, median age, and cumulative degree days above 0 °C, an optimal threshold of 3.32 crowdsourced tick submissions allowed the detection of CSDs with established tick populations while maximizing sensitivity and specificity (both 0.76).

**Conclusions:**

This work introduces a framework for analyzing crowdsourced surveillance data by correcting for participation bias and benchmarking against active surveillance. Unlike approaches relying on traditional or unadjusted data, it incorporates socioecological factors associated with reporting. We show that crowdsourced data can yield reliable inferences about vector distributions and provide a methodological approach that could be applied across species and regions. Applied to eTick, it supports integration into surveillance systems to improve early detection, targeting interventions, and cost-effective monitoring of emerging risks by public health.

## Introduction

Tick-borne diseases represent a growing public health concern in Canada. In recent decades, several tick species have expanded their geographic ranges and population sizes, largely driven by climate and land use changes [1,2]. Notably, *Ixodes scapularis*, the primary vector of the agent of Lyme disease (LD), the most commonly reported vector-borne illness in Canada, has seen significant range expansion [3-5]. Geographic ranges of other species transmitting various pathogens, such as *Ixodes cookei* and *Dermacentor* spp., are also expanding, thereby increasing current and future risks of tick-borne diseases in Canada [1,6,7]. In this context, national and provincial public health authorities across Canada have implemented surveillance programs to identify where tick populations are spreading and to monitor disease incidence trends. These efforts identify where tick-borne disease risk is occurring, allowing targeted promotion of public awareness of risk and prevention methods and providing guidance to health care professionals [8]. However, tick presence and disease risk vary regionally due to ecological and environmental factors, including the availability of suitable hosts for ticks and pathogens, climate and habitat suitability for tick survival, and human exposure [1,9]. Canada’s vast territory and diverse environments present major challenges for conducting consistent nationwide tick surveillance [10]. Tick-borne diseases demonstrate the importance of a One Health approach, as disease risk emerges from interactions among environmental conditions, animal hosts, and human behavior. While tick occurrence depends on climate, habitat, and wildlife hosts, exposure of humans and domestic animals is shaped by patterns of outdoor activity and contact with suitable tick habitat [11]. Domestic animals, which are frequently exposed to ticks, can serve as sentinels of local tick presence, underscoring the value of integrated surveillance approaches that integrate data from multiple sources (ie, humans, pets, and wildlife) and the environment [11-14].

Traditional methods for tick surveillance include active and passive surveillance conducted by public health organizations. In active surveillance, field teams perform standardized environmental sampling, generally involving drag sampling to collect questing ticks [10]. This approach provides strong scientific rigor because it allows the collection of tick abundance data, ensuring repeatability and comparability across sites due to standardized protocols for sampling and site selection [15,16]. However, this approach is also labor-intensive, and its inherent high costs limit its use geographically and temporally [17]. In passive surveillance, wider geographic coverage is possible at a lower cost, as ticks are voluntarily submitted to public health laboratories by the general population via health care professionals, medical clinics, or veterinary clinics. These laboratories undertake tick species identification and molecular screening for targeted pathogens [18]. Historically, passive surveillance has played a central role in detecting the emergence of new tick populations in Canada, providing early warning signals [18-20]. However, it has been gradually phased out in most regions across Canada due to insufficient laboratory resources to handle the increasing submission volumes associated with both higher numbers of ticks in endemic regions and expanding tick species distributions [16,21,22]. Consequently, an alternative passive surveillance framework is needed to ensure continued monitoring of emerging tick species and tick-borne diseases and to enable long-term data collection critical for understanding the spread of tick-borne diseases [23].

Citizen science approaches such as crowdsourcing-based data are increasingly used to complement field observations to estimate species distributions [24]. Crowdsourcing-based tick data collection is an emerging approach that engages the public to contribute to monitoring efforts via web platforms and applications [25]. Numerous examples from Europe and North America demonstrate the utility of such participatory approaches in complementing traditional tick surveillance methods [26-30]. The benefits of these approaches for public health include access to large volumes of data, broad geographic coverage, timely observations, and increased public engagement and risk awareness [31]. In 2017, eTick was launched as the first crowdsourcing platform dedicated to tick data collection in Canada. The general public, veterinarians, and health care professionals are invited to submit digital images of ticks found on people, on animals, or in the environment for free professional identification using a validated method [32]. When submitting a tick, users mark the location on a map to identify the presumed location of acquisition and provide additional information about recent travel. Submitters receive tick species identification results along with health prevention recommendations within 2 business days, and the municipalities where ticks were found are displayed on a publicly accessible map.

Multiple factors are thought to influence the data structure in citizen science and passive tick surveillance initiatives and must be identified and accounted for if the data are to be unbiased and useful for driving public health actions. Participation varies with age and sex [33-36], personal values, intrinsic interest, and a sense of belonging to a community [31,37-39], as well as education, income, and ethnicity, which may either encourage participation through increased awareness and risk perception or represent barriers to participation [31,34,36,37,39-41]. In tick-borne disease initiatives, pet owners are typically the most represented group, along with people living in houses with yards and those regularly doing outdoor recreational activities [35].

Geographical biases in sampling are a common issue with crowdsourced data. Areas that are more accessible and that offer higher recreational interest, such as public parks and protected areas, may yield more observations [42,43]. Most tick submissions tend to be associated with peridomestic exposures [44], but these are influenced by the degree with which human settlement is juxtaposed with wildland [45]. The geographic scope of tick surveillance is limited by the geographic scope of the human populations. Tick submission numbers are proportional to the size of the participating human population and must be accounted for in data analysis [18-20,40,41]. Furthermore, expectations of spatial accuracy of tick encounter locations identified in passive surveillance must be realistic, as ticks can remain attached to their hosts for several days and often go unnoticed [21,46]. If possible, tick data from submitters with a recent history of long-distance travel should be eliminated from analysis [47].

A significant potential weakness of crowdsourced data is that, for some species, individual specimens may be found outside their geographic range due to their dispersion by migratory animals [48]. Crowdsourced data from these species require careful analysis and processing to avoid overestimating geographic ranges and ecological niches. For many tick species, ticks found by the public do not necessarily indicate locations where annually reproducing tick populations have become established. Many tick species are dispersed over long distances from their source populations by hosts (particularly migratory birds), and these ticks can survive to molt and feed as the next instar on hosts, including humans and their pets [48]. Locations where submitted ticks comprise only these “adventitious ticks” must be distinguished from locations with established tick populations [20]. The primary method for making this distinction involves determining a threshold number of submitted ticks per geographic unit based on statistical comparisons of the submission rates from locations with and without established populations identified through “gold standard” active surveillance [20]. Including environmental data that determine where tick populations can persist (habitat, host densities, and climate [1,9,49,50]) in analyses of passive surveillance data can help discriminate ticks coming from established populations versus adventitious ticks [20].

This study aimed to develop robust, evidence-based indicators for assessing the risk of tick population establishment using crowdsourced tick surveillance data. Specifically, our objectives were to (1) characterize spatial patterns in eTick submission rates and identify socioecological factors influencing their occurrence and frequency and (2) assess the ability of indicators derived from eTick data to predict census subdivisions (CSDs) with established tick populations. We focused on *I scapularis* in Quebec, Canada, where long-term active surveillance provides a reliable benchmark for validation. By leveraging Quebec’s extensive dataset, this study generated practical, scalable insights to improve tick surveillance and risk assessment frameworks across Canada and in comparable international contexts.

## Methods

### Study Design and Setting

In this study, we conducted a retrospective ecological assessment for the effectiveness of crowdsourced data to detect established tick populations in Quebec, Canada, from 2020 to 2023. Here, an established tick population refers to a locally reproducing population considered persistent over time. The criteria used to infer establishment followed the *Institut national de santé publique du Québec’s* (INSPQ) risk classification approach [51], itself based on [52] (see section Active Tick Surveillance for details). The unit of analysis was the municipality, defined as CSDs, totaling 1282 across the province [53], representing the finest spatial scale at which all relevant data were available. Approximately 80% of the province’s population of 8 million lives in metropolitan areas, concentrated in the south, with the most populated being Montréal, Québec City, and Sherbrooke [54]. The province has seen ticks and LD emerging gradually over the last two decades. The most endemic regions, with the longest history of emergence, are the southernmost regions of Montérégie and Estrie, near the US border [55,56]. Since initial emergence, endemic regions have progressively expanded across southern Quebec, and human LD cases have been constantly rising. The dominant forest types are deciduous and mixed forests, and the potential exposure to ticks varies depending on land use and human activities, ranging from peridomestic and rural environments to recreational areas, such as natural parks in urban settings.

### Data Sources and Variable Construction

#### Active Tick Surveillance

Active surveillance data were obtained from the INSPQ for 2014 to 2023. The INSPQ is Quebec’s public health expertise and reference center, playing a key role in the surveillance and prevention of LD and other tick-borne diseases. Active surveillance was conducted at sampling sites located in public nature parks or on private lands with owner authorization. Site selection was based on habitat suitability for ticks (eg, deciduous or mixed forests) and signals of emerging risk, such as reported human LD cases or *I scapularis* collected through active and passive surveillance. Sampling took place between May and August under conditions without rain and with dry soil. At each site, a standardized drag sampling protocol was used. A team of 2 field technicians sampled each site, using a 1-m² flannel cloth dragged horizontally along 2 transects: one along the trail edge and one 25 m into the forest, parallel to the trail. Each technician sampled 1000 m², for a total sampled area of 2000 m² per site. The flannel cloths were inspected every 25 m for ticks. Ticks were removed with tweezers, placed in tubes containing 70% ethanol, and sent to the Quebec Public Health Laboratory (*Laboratoire de santé publique du Québec*) for species identification using taxonomic keys and pathogen testing [57]. Counts of the 3 life stages of *I scapularis* (larvae, nymphs, and adults) were calculated for each site visit and used to classify CSDs as having established tick populations or not. A CSD was considered established if at least six ticks of any life stage or at least two different life stages were collected within the same year in the municipality. If the indicator was met for at least 1 year during our study period, the municipality was considered as having an established *I scapularis* population [52,58].

#### Crowdsourced Tick Surveillance

Crowdsourced tick surveillance data were obtained from the eTick platform. We extracted all submissions identified as *I scapularis* for 2020 to 2023 in Quebec. Although eTick was launched in 2017, this time frame was considered representative of a stable period for analysis, following an initial phase of increasing familiarity and adoption of the eTick platform by the public. Retrospective analysis of the number of tick submissions suggested that the COVID-19 pandemic did not have a large effect on submissions, possibly due to limited restrictions on peridomestic activity and outdoor pursuits. Furthermore, the launch of the mobile eTick application in early 2020, just prior to lockdown, likely facilitated engagement and sustained user participation.

Data reported by users for each submission included the geographic location where the tick was found (ie, users marked the presumed location of acquisition on a zoomable map, from which the research team extracted coordinates), the date it was found, and whether the tick was found attached to an animal, attached to a human, or unattached (eg, crawling on a host or free in the environment), which is referred to as the origin of the tick throughout the paper. Additionally, for cases where the tick was attached to a human, users reported the age and gender of the individual. Users also reported whether they had traveled outside their municipality within 2 weeks prior to finding the tick. Starting in 2023, data collection also included information from the submitting individual regarding their profession (health care professional, veterinarian, or general public participant). Finally, additional details were provided by eTick’s professional entomologist team: the tick life stage and sex, and unique unidentifiable user IDs. A public map is available on the platform, displaying tick submission locations, along with the tick species, locality, date of observation, origin, and tick pictures. All other information provided by users remains confidential.

The total number of individual submissions per user was calculated, and the distribution of the number of submissions per user was inspected graphically to detect potential outliers (ie, superusers). Superusers were defined a priori as users with submission counts exceeding the 99.9th percentile of the distribution.

Total tick submissions by origin type and overall (including unknown origins) were summed at the CSD level, given submission locations found within CSD boundaries. These counts were calculated both with and without the superuser outliers. Ticks that could have been acquired during travel were excluded from the analysis to reduce potential bias from submissions not representing the geographic location of tick exposure [19,20].

#### Socioecological Factors

Here, socioecological factors refer to demographic and socioeconomic characteristics that may influence community participation and environmental conditions known to affect tick populations. Corresponding variables were constructed and considered as predictors or confounders in the statistical models described below (see section Analysis for Details).

CSD statistics for human population size, median age, and median household income were obtained from the 2021 census [53,59]. All monetary values are reported in Canadian dollars. The conversion rate at the time of the study was CAD $1=US $0.72. Missing data occurred for these variables at the CSD level, primarily due to census data suppression or nonrelease resulting from confidentiality rules, low population counts, or high nonresponse rates [60]. These missing data largely occurred in remote or Indigenous CSDs, which represent subpopulations that differ from the population relevant to this study (ie, potential users of the eTick platform). CSDs with missing values for any of these variables were therefore not included in the analysis (194 CSDs excluded, representing 15% of Quebec’s 1282 CSDs).

A variable derived from Google Trends was used to account for CSD-level participation toward eTick [61,62]. Google Trends is an open-access tool to extract information about Google search trends [63]. We assumed that Google searches that included keywords “ticks” and “Lyme disease,” in either English or French, represented interest in ticks and LD, and thus served as a proxy to contribute to eTick. Google Trends data for the study variable were extracted at the municipal level for 2020 to 2023. Municipalities were then matched to their corresponding CSD based on name. The returned data use a relative scale from 0 to 100, where 100 represents the highest proportion of keyword searches relative to the total searches in a given region and period, and 0 represents the lowest level of interest [64].

A variable representing human exposure to tick habitats was derived as the proportion of interface zones between human settlements and wildlands. Data from the global wildland-urban interface (WUI) [65] were used to compute the WUI proportion per CSD. The calculation included all WUI categories dominated by forest, shrub, and wetland types, including both intermix and interface zones. The full methodology used to produce this layer has been published [65]. In brief, the WUI classifies areas into 2 categories: intermix and interface. The intermix WUI has ≥6.17 buildings per km² and ≥50% wildland vegetation. The interface WUI has ≥6.17 buildings per km² and <50% wildland vegetation but is located within 2.4 km of a large patch (≥5 km²) of wildland with >75% vegetation cover.

Finally, to compute annual values of cumulative degree days (DD) >0  °C, we used the Daymet dataset [66], which provides daily meteorological data at a 1 km spatial resolution across North America by integrating data from weather stations and other supporting sources through interpolation methods. This variable served as a proxy for 2 factors. First, the yearly exposure duration to host-seeking ticks [41], and second, a determinant of the potential for tick population establishment, given an approximate constraint of 2843 DD >0 °C to maintain populations [67,68]. Yearly CSD values were calculated as the mean 1-km resolution of pixel values whose centroids fell within CSD boundaries.

### Ethical Considerations

The eTick platform collects routine passive surveillance data on ticks in Canada. On the basis of the nature of the data collected and the collection process, and acknowledging that no formal research question was specifically formulated to acquire these data, the Bishop’s University Research Ethics Board stated that no ethical review is required for data collection through the eTick platform (Multimedia Appendix 1). Here, we make a secondary use of nonidentifiable data; individuals cannot be identified from any images or materials in the paper or supplementary files, a case defined in the Canadian research ethics guidelines for research involving humans (TCPS 2) as not requiring ethical review [69].

Before submission, all eTick users must consent to the use of their deidentified data for surveillance purposes and for future unspecified research [70]. Personal information (name, username, email address) is collected exclusively for communication purposes and visible only to site administrators. Data extracts used for surveillance or research do not include personal information in their data fields. No compensation was provided to participants.

### Analysis

All analyses were conducted in R version 4.5.1 (R Foundation for Statistical Computing) [71].

#### Objective 1: Spatial Patterns and Socioecological Factors Associated With eTick Submissions

##### Assessing for Clusters in Tick Submissions

Spatial patterns in tick submissions were analyzed separately for tick submissions originating from humans and animals. The total CSD-level submissions for both types of submissions over the study period were visualized as rates normalized by CSD population size. Rates were calculated as: (number of submissions in the CSD/CSD human population)×100,000 and then mapped using QGIS (version 3.44.1).

The spatial scan statistic assessed for significantly high rates in the number of submissions at the CSD level [72]. CSD locations were represented by the centroid coordinates. The method used a circular scanning window that moved across the study area, varying in size from 0% to 50% of the study population, to identify potential windows in which submission rates are significantly higher than in surrounding areas [73]. Prior to the scan, overdispersion in submission counts was assessed using the Dean test [74]. As significant overdispersion was present, spatial scan analyses were conducted under a negative binomial distribution, implemented in the *DCluster* package in R [75]. As shown by Loh and Zhu [76], this approach is appropriate for overdispersed count data and reduces the risk of detecting false-positive clusters driven by extravariability rather than true spatial clustering [73]. The statistical significance of each potential cluster was evaluated using Monte Carlo simulation with 999 random permutations. Clusters were considered statistically significant at *P*≥.05. The spatial scan analysis was performed separately for human- and animal-origin data.

##### Identifying Socioecological Factors Associated With Tick Submission Occurrence and Counts

We assessed for socioecological factors associated with the occurrence and counts of tick submissions, at the CSD-year levels, using zero-inflated regression models.

Logarithmic transformations were first applied to normalize skewed predictor variable distributions, then all predictors were centered and scaled to ensure appropriate model behavior (ie, reduce multicollinearity, help produce reliable model estimates, and facilitate interpretation of model estimates). Collinearity among variables was verified using Pearson correlation coefficients and the variance inflation factor (using maximal thresholds of 0.7 and 3, respectively [77]). Significant overdispersion was detected based on the dispersion ratio, and the presence of excess zeros was confirmed through the Vuong test.

A zero-inflated negative binomial model was fitted using the *pscl* package, version 1.5.9 [78]. These models have 2 distinct components: a logistic component that models the probability of having structural zeros (ie, populations not at risk; in our case, regions unsuitable for the presence of tick populations) and a count component that models the number of events (including zero counts) in the at-risk population (in our case, regions suitable for tick presence, where tick submissions may or may not occur). A backward stepwise selection procedure was used for final variable selection [79].

Model fit was assessed through graphical inspection of the residuals. Cook distance was calculated to identify potential outliers with a threshold of 1. When outliers were detected, models were refitted excluding the outlier CSDs. Observations were considered influential if their associated difference in beta exceeded the conventional threshold (2/√n) for any key coefficient or if their removal altered the statistical significance of predictors [80]; such CSDs were excluded; otherwise, they were retained. The presence of residual spatial autocorrelation, which could bias the results, was assessed using the global Moran I test on standardized Pearson residuals with the *ape* package in R.

### Objective 2: Ability of eTick Indicators for Detecting Established Tick Populations

#### Detecting Established Tick Populations

Logistic regression models were used to assess the value of using eTick surveillance data to predict the presence of an established tick population in a CSD. Observed tick establishment status was determined using INSPQ active surveillance data, following the criteria described in section Active Tick Surveillance, and including CSDs with at least one active surveillance visit since 2014 (n=343). Model building was conducted separately for 3 types of eTick indicators: number of submissions of (A) human origin, (B) animal origin, and (C) all origins combined. Three model structures were assessed for each indicator: (1) a global model including the respective eTick indicator and all socioecological factors identified as significant in objective 1 (models A1, B1, and C1), (2) a reduced model including the eTick indicator and CSD population size (models A2, B2, and C2), and (3) a basic model including only the eTick indicator (models A3, B3, and C3).

We used the same variable data transformation and model building approaches as described for objective 1. Backward stepwise selection based on Akaike information criterion was used to identify the best model for the global model structures. We compared the explanatory power of the nested models using the likelihood ratio test, and assessed model performance using area under the curve (AUC) and Akaike information criterion. Finally, model fit was evaluated using the Hosmer-Lemeshow test, along with graphical inspection and outlier detection as described earlier in objective 1.

#### Identifying Thresholds of Submissions Optimizing Tick Population Detection Accuracy

Thresholds for eTick submission data were calculated to maximize sensitivity, specificity, or a balance between both metrics for detecting established tick populations. Analyses focused on submissions from all origins, with thresholds derived from the linear predictors of models C1, C2, and C3. Optimal cutoffs for the linear predictors were determined using the *OptimalCutpoints* package in R [81]. The first 2 methods maximize sensitivity and specificity separately, while maintaining the other parameter at a minimum threshold [82-84]. For instance, in the method optimizing sensitivity, we set specificity to 0.5 and maximized sensitivity under this condition. The third method sought a balance between sensitivity and specificity by minimizing the absolute difference between them [85-87].

To facilitate interpretation of the resulting linear predictor thresholds, illustrative scenarios were subsequently developed. These scenarios were not intended for inference, but rather to translate the model-derived thresholds into interpretable submission counts, by fixing covariates at representative values reflecting different socioecological contexts within the study area. To this end, CSDs were grouped according to the 2 most influential covariates affecting the relationship between submissions and tick population presence: climate suitability and human population size. CSDs were first classified according to their climatic suitability for tick establishment, using the threshold of 2843 DD>0 °C [67]. Within climatically suitable CSDs, a population threshold of 100,000 inhabitants was applied to distinguish large population centers from medium and small population centers [88]. As climatically unsuitable CSDs generally have smaller populations, we used the small population center threshold (30,000 inhabitants [88]) in this group to improve descriptive stratification. In each group, the number of submissions corresponding to each optimized cutoff of the linear predictor was calculated while holding model covariates at their mean values.

## Results

### Objective 1: Spatial Patterns and Socioecological Factors Associated With eTick Submissions

#### Characteristics and Spatial Patterns of Tick Submissions

A total of 2668 photographic submissions to eTick were identified as *I scapularis* collected in Quebec during the study period, including 429 (16.1%) submissions in 2020, 722 (27.1%) in 2021, 855 (32.1%) in 2022, and 662 (24.8%) in 2023.

Of these, 313 (11.7%) submissions were excluded from the analysis because users reported travel outside their municipality of residence within the 2 weeks prior to submission.

Among the 2355 included submissions, most (n=1594, 67.7%) submitted ticks were found attached to animals, whereas 28.6% (n=673) were found attached to humans and 3.7% (n=88) were found from the environment (Table 1). Most submitted ticks were adult females (1639/1955, 83.8%), followed by adult males (270/1955, 13.8%) and nymphs (46/1955, 2.4%). The life stage could not be distinguished for 400 (17%) of 2355 submissions. Among submissions with reported submitter profession (this question was introduced in 2023 and was therefore missing for 1,524/2355, 64.7%, submissions), most were submitted by members of the public (719/831, 86.5%), with smaller proportions of submissions from veterinary professionals and human health care professionals (Table 1).

**Table 1.** Distribution of blacklegged tick (*Ixodes scapularis*) submissions reported through the eTick crowdsourcing passive surveillance platform in Quebec, Canada, between 2020 and 2023, categorized by tick, host, and submitter characteristics^a^.

Variables and characteristics		Submissions, n (%)
Tick stage and sex^b^ (n=1955)		
Nymph		46 (2.4)
Adult (male)		270 (13.8)
Adult (female)		1639 (83.8)
Origin of the tick (n=2355)		
On a human		673 (28.6)
On an animal		1594 (67.7)
In the environment		88 (3.7)
Age of the human host (y; n=671)^c^		
0‐20		185 (25.6)
21‐40		131 (19.5)
41‐60		229 (34.1)
≥61		126 (18.8)
Submitter profession^d^ (n=831)		
Member of the public		719 (86.5)
Veterinary professional		101 (12.2)
Human health care professional		11 (1.3)

a Only considering the submissions for which the user did not report travel history within the previous 2 weeks (n=2355).

b Was missing in 400 (17%) of 2355 cases because not all ticks could be identified to a life stage from the submitted picture.

c Was missing in 1684 (71.5%) of 2355 cases because not all users reported this information.

d Was missing in 1524 (64.7%) of 2355 cases because it has been only added to the eTick questionnaire in 2023. For users who submitted before 2023 and later returned, their profession was retrieved and added to their earlier submissions.

Most (1600/1789, 89.4%) users made only one submission to the platform, with an average of 1.49 (SD 7.38) submissions per user. Among users who made multiple submissions, 2 were outliers regarding submission frequency (submitting 137 and 264 times) and were therefore excluded from the subsequent analysis. After removing these outliers, the average number of submissions per user was 1.27 (SD 2.38), and the number of submissions ranged from 0 to 148 per CSD, with a mean of 1.56 (SD 6.82) submissions per CSD.

Of the 1282 CSDs, 454 (35.4%) had at least one tick submission. Among these tick-positive CSDs, 210 (46.3%) were identified exclusively through submissions of ticks found attached to animals, 85 (18.7%) were identified through submissions of ticks found attached to humans, and 150 (33%) were identified through both categories of submissions. Strong overdispersion was detected in CSD-level submission counts for both human and animal-origin ticks (Dean test, *P*<.001). Spatial scan analyses accounting for overdispersion did not identify any statistically significant spatial clusters for either human- or animal-origin submissions. Spatial patterns are therefore described qualitatively.

**Figure 1.**
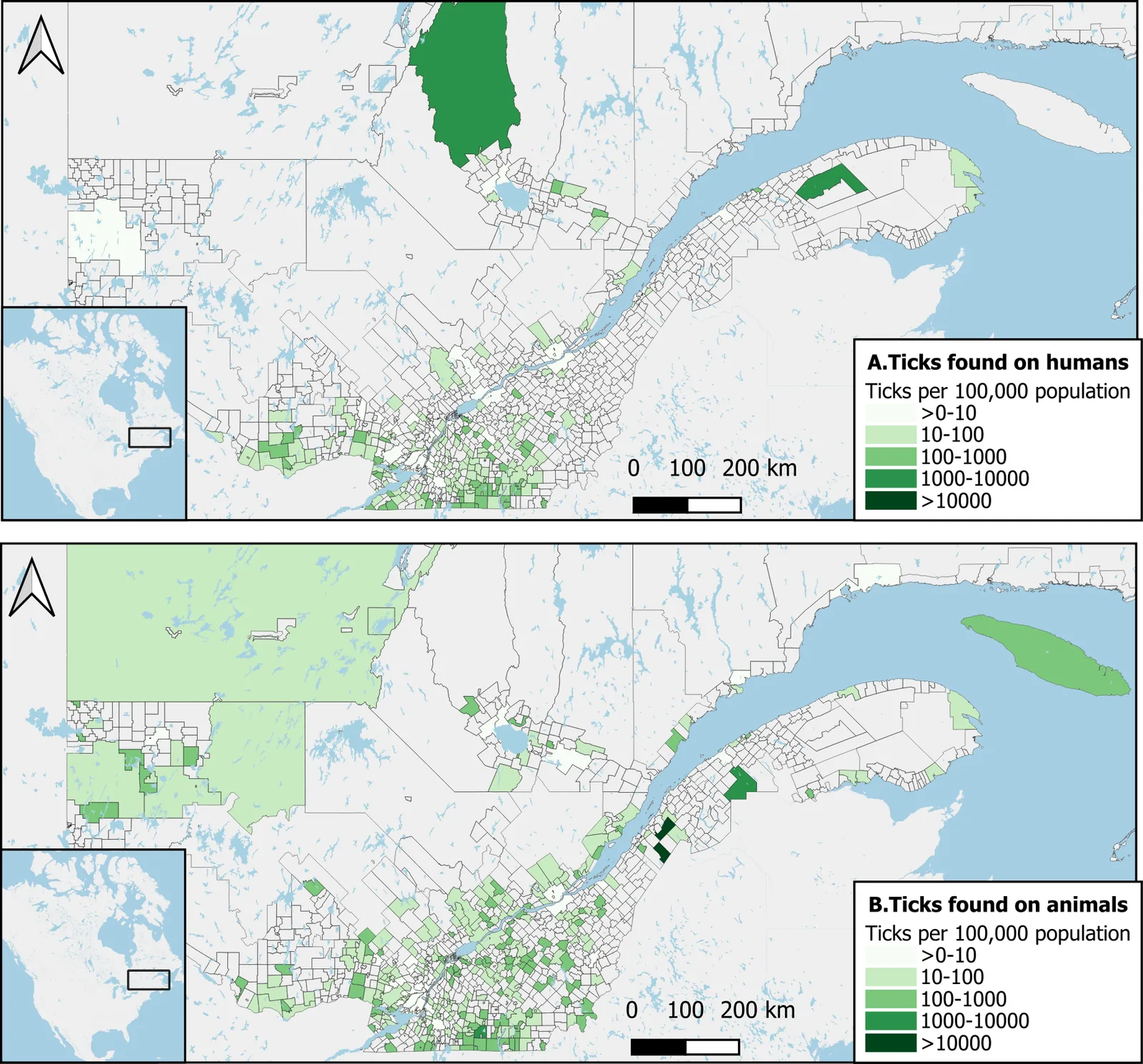
Blacklegged tick (*Ixodes scapularis*) submission rates reported through the eTick crowdsourcing passive surveillance platform, in Quebec, Canada, from 2020 to 2023, showing the spatial distribution of ticks found attached to (A) humans and (B) animals at the census subdivision level.

For both categories, the submissions were geographically distributed across municipalities in southern Quebec (Figure 1). Submissions of ticks found attached to humans accounted for 29% of all submissions and were predominantly reported from the southern regions of Quebec, particularly Estrie, Outaouais, and Montérégie. Additional areas with higher submission rates for human-origin ticks were observed in Centre-du-Québec and Chaudière-Appalaches. Ticks found attached to animals represented 68% of all submissions and showed a broader geographic distribution. Additionally, higher submission rates for animal-origin ticks were observed in more northerly regions, including Abitibi-Témiscamingue, Mauricie, Capitale-Nationale, and Centre-du-Québec.

#### Socioecological Factors Associated With Tick Submissions

The human population size of CSDs was the strongest predictor of both the likelihood (Table 2) of tick submissions and their count (Table 3). Each unit increase in the log-transformed population was associated with a 2.04-fold increase in submission counts (incidence risk ratio [IRR] 2.04, 95% CI 1.86‐2.25; *P<.*001) and a reduction in the odds of structural zeros (odds ratio [OR] 0.13, 95% CI 0.05‐0.31; *P<.*001). The log-transformed proportion of WUI zones significantly influenced both components of the model. CSDs with higher WUI proportions had more submissions (IRR 1.43, 95% CI 1.26‐1.63; *P<.*001), but they were also associated with greater odds of being a structural zero (OR 2.65, 95% CI 1.21‐5.79; *P*=.01). The number of DD>0 °C significantly reduced the odds of structural zeros (OR 0.10, 95% CI 0.04‐0.24; *P<.*001). Socioeconomic factors also positively influenced submission counts. Higher median household income and median age were associated with increases in submissions (IRR 1.35, 95% CI 1.20‐1.52; *P<.*001; and IRR 1.45, 95% CI 1.26‐1.67; *P<.*001, respectively). One CSD, Lac-Brome in the Estrie region, was identified as an outlier for submission counts (n=148) and excluded from the model fit. No residual spatial autocorrelation was detected (*P*=.60), supporting the robustness of the model.

**Table 2.** Factors associated with the occurrence of blacklegged tick (*Ixodes scapularis*) submissions, reported through the eTick crowdsourcing passive surveillance platform across census subdivisions (CSDs) in Quebec, Canada, from 2020 to 2023, according to the zero-inflation component of a zero-inflated negative binomial model

Effect^a^	Estimate	SE	*P* value	OR^b^ (95% CI)
Human population^c^	−2.08	0.50	<.001	0.13 (0.05‐0.31)
Proportion of WUI^c^^,d^	0.97	0.40	.02	2.65 (1.21‐5.79)
Annual cumulated Degree Days >0 °C	−2.39	0.48	<.001	0.10 (0.04‐0.24)

a All the variables were centered and scaled before the analysis.

b OR: odds ratio.

c Logged variable.

d WUI: wildland-urban interface.

**Table 3.** Factors associated with the abundance of blacklegged tick (*Ixodes scapularis*) submissions, reported through the eTick crowdsourcing passive surveillance platform across census subdivisions (CSDs) in Quebec, Canada, from 2020 to 2023, according to the count component of a zero-inflated negative binomial model (n=1067).

Effect^a^	Estimate	SE	*P* value	IRR^b^ (95% CI)
Google Trends index	0.08	0.04	.03	1.08 (1.01‐1.16)
Median income	0.30	0.06	<.001	1.35 (1.20‐1.52)
Median age	0.37	0.07	<.001	1.45 (1.26‐1.67)
Human population^c^	0.71	0.05	<.001	2.04 (1.86‐2.25)
Proportion of WUI^c^^,d^	0.36	0.07	<.001	1.43 (1.26‐1.63)

a All the variables were centered and scaled before the analysis.

b IRR: incidence risk ratio.

c Logged variable.

d WUI: wildland-urban interface.

### Objective 2: Ability of eTick Indicators for Detecting Established Tick Populations

#### The Detection of Established Blacklegged Tick Populations

In all 3 sets of models, the more complex models that incorporated socioecological factors outperformed the simpler models, which included submissions with population size or submissions alone (Table 4). The model using submissions of ticks found attached to humans and socioecological covariates (model A1) performed best among all models (AUC 0.86). The model using all types of submissions (model C1) had lower performance (AUC 0.85) compared to the model using submissions of ticks found attached to humans only (model A1), followed by the model using submissions of ticks found attached to animals only (model B1, AUC 0.83).

**Table 4.** Performance of logistic regression models predicting the presence of established blacklegged tick (*Ixodes scapularis*) populations at the census subdivision (CSD) level in Quebec, Canada, between 2020 and 2023, using eTick crowdsourced surveillance indicators^a^.

Model^b^	Set of covariables included for each candidate model	AIC^c^	ΔAIC^d^	AUC^e^
A1	Submissions of ticks found attached to humans, population size, income, age, proportion of WUI^f^, and DD^g^ >0 °C	289.24	0.00	0.86
C1	All tick submissions, population size, income, age, and DD >0 °C	295.82	6.58	0.85
B1	Submissions of ticks found attached to animals, population size, income, age, and DD >0 °C	312.26	23.02	0.83
A2	Submissions of ticks found attached to humans and population size	366.99	77.75	0.70
A3	Submissions of ticks found attached to humans	370.23	80.99	0.67
C2	All tick submissions and population size	379.54	90.31	0.68
C3	All tick submissions	384.70	95.46	0.64
B2	Submissions of ticks found attached to animals and population size	396.15	106.91	0.61
B3	Submissions of ticks found attached to animals	396.17	106.93	0.59
0	Intercept only	409	119.77	0.50

a Accounting or not for confounding variables identified in the objective.

b Models are built separately for 3 types of eTick indicators: the number of submissions of (A) human origin, (B) animal origin, and (C) all origins combined and are compared across 3 levels of model complexity.

c AIC: Akaike information criterion.

d Differences in AIC (ΔAIC) represent the comparison for all models against the top model, A1.

e AUC: area under the curve.

f WUI: wildland-urban interface.

g DD: degree days.

The number of human-attached submissions to eTick was significantly associated (OR 3.34, 95% CI 2.30‐4.86; *P<.*001; Table 5) with an increased likelihood of finding established tick populations through active surveillance. Socioecological factors influencing tick presence or population participation in crowdsourced surveillance improved model performance. Positive effects were observed for the log DD >0 °C (OR 4.73, 95% CI 2.97‐7.52; *P<.*001) and for the proportion of WUI (OR 1.37, 95% CI 1.03‐1.83; *P*=.03). Negative effects were observed for the log of population size (OR 0.37, 95% CI 0.24‐0.56; *P<.*001), median income (OR 0.42, 95% CI 0.28‐0.63; *P<.*001), and median age (OR 0.62, 95% CI 0.42‐0.92; *P*=.02).

**Table 5.** Outputs of the best logistic regression models predicting the presence of established blacklegged tick (*Ixodes scapularis*) populations at the census subdivision (CSD) level in Quebec, Canada, between 2020 and 2023, using eTick crowdsourced surveillance indicators and socioecological factors^a^.

Variable^b^	OR^c^ (95% CI)	*P* value
A1. Best model using submissions of ticks found attached to humans and socioecological covariables		
Number of submissions found attached to humans^d^	3.34 (2.30‐4.86)	<.001
Population size^d^	0.37 (0.24‐0.55)	<.001
Median income	0.42 (0.28‐0.63)	<.001
Median age	0.62 (0.42‐0.92)	.02
Proportion of WUI^e^	1.37 (1.03‐1.83)	.03
DD^f^ >0 °C	4.73 (2.97‐7.52)	<.001
B1. Best model using submissions of ticks found attached to animals and socioecological covariables		
Number of submissions found attached to animals^d^	2.90 (1.93‐4.36)	<.001
Population size^d^	0.36 (0.23‐0.56)	<.001
Median income	0.50 (0.35‐0.73)	<.001
Median age	0.67 (0.46‐0.97)	.03
DD >0 °C	4.67 (3.04‐7.18)	<.001
C1. Best model using all tick submissions and socioecological covariables		
Number of submissions^d^	3.86 (2.51‐5.93)	<.001
Population size^d^	0.31 (0.20‐0.48)	<.001
Median income	0.45 (0.30‐0.66)	<.001
Median age	0.63 (0.43‐0.92)	.017
DD >0 °C	4.78 (3.05‐7.50)	<.001

a Models are built separately for 3 types of eTick indicators: the number of submissions of (A) human origin, (B) animal origin, and (C) all origins combined.

b All variables were scaled prior to the analysis.

c OR: odds ratio.

d Log-transformed variable.

e WUI: wildland-urban interface.

f DD: degree days.

#### Thresholds of Submissions Optimizing Tick Population Detection Accuracy

Thresholds of tick submissions calculated using the linear predictor from the most complex model (C1) outperformed those from the simpler models across all optimization methods. A cutoff of −2.30 optimized sensitivity at 0.96, while a cutoff of 0.26 optimized specificity at 0.91 (Table 6). These optimizations were achieved while ensuring that the opposite parameter did not fall below 0.50. When optimizing both parameters simultaneously, sensitivity and specificity were balanced at 0.76 with a cutoff of −0.66. The method that minimized the overall error rate was the one optimizing specificity, resulting in an error rate of 0.22.

**Table 6.** Cutpoints of linear predictors optimizing sensitivity, specificity, or the equilibrium between both for detecting established tick populations at the census subdivision level in Quebec, Canada, between 2020 and 2023, according to the best-performing model using the eTick crowdsourced surveillance indicator, including all origins combined.

Model and method of cutpoint optimization	Sensitivity (%)	Specificity (%)	Error rate (%)	Linear predictor cutpoint (submissions)
Optimization of sensitivity^a^				
C3. Model using all submissions	0.55	0.61	0.41	−0.87
C2. Model using all submissions and population size	0.72	0.53	0.42	−1.18
C1. Model using all submissions, population size, income, age, and DD^b^ >0 °C	0.96	0.51	0.36	−2.30
Optimization of specificity^c^				
C3. Model using all submissions	0.55	0.61	0.41	−0.81
C2. Model using all submissions and population size	0.5	0.86	0.25	−0.50
C1. Model using all submissions, population size, income, age, and DD >0 °C	0.5	0.91	0.22	0.26
Optimization of the balance between sensitivity and specificity				
C3. Model using all submissions	0.55	0.61	0.41	−0.81
C2. Model using all submissions and population size	0.63	0.63	0.37	−0.90
C1. Model using all submissions, population size, income, age, and DD >0 °C	0.76	0.76	0.24	−0.66

a The minimum value tolerance for specificity is 0.50.

b DD: degree days.

c The minimum value tolerance for sensitivity is 0.50.

The CSDs grouped by climatic suitability and human population showed diverse environmental and demographic characteristics (Table 7). Across the study region, for a typical area with an average DD >0 °C of 3199 and an average human population of 19,838, the optimal submission threshold for detecting an established tick population—balancing sensitivity and specificity—is estimated at 3.32. This threshold increases to 7.54 when optimizing specificity and decreases to 0.51 (rounded to 1 submission in practice) when optimizing sensitivity. When analyzing CSDs by group, climatically unsuitable medium population centers had the highest threshold values, followed by climatically suitable large population centers, climatically unsuitable small population centers, and, finally, climatically suitable small and medium population centers.

**Table 7.** Optimal number of submissions (cutpoints) for optimizing sensitivity, specificity, or both, based on the top-performing model (C1) predicting established blacklegged tick (*Ixodes scapularis*) populations at the census subdivision (CSD) level in Quebec, Canada, between 2020 and 2023, using the eTick crowdsourced surveillance indicator including all origins combined and socioecological factors^a^.

CSD group	Average covariate values across the CSD group				Optimal number of submissions (tick count) according to each cutpoint optimization method		
	DD >0 °C	Population (n)	Median income (CAD $)	Median age (y)	Sensitivity≈specificity	↑ Specificity	↑ Sensitivity
Overall average	3199	19,838	75,384	47.5	3.32	7.54	0.51
Climatically suitable large population centers (>2843 DD^b^ >0 °C; ≥100,000 population; n=9)	3603	430,893	74,933	42.2	12	24.67	3.55
Climatically suitable small and medium population centers (>2843 DD >0 °C; <10,000 population; n=247)	3344	9202	77,717	46.4	1.92	4.76	0.02
Climatically unsuitable medium population centers (<2843 DD >0 °C; ≥30,000 population; n=3)	2769	97,172	61,300	50.2	64.40	128.16	21.90
Climatically unsuitable small population centers (<2843 DD>0 °C; <30,000 population; n=74)	2684	3478	68,129	51.7	11.87	24.41	3.51

a For ease of interpretation, the cutpoints are calculated for typical CSDs grouped by climate suitability and population size, with demographics and environmental covariates fixed at their mean values within each CSD group.

b DD: degree days.

**Figure 2.**
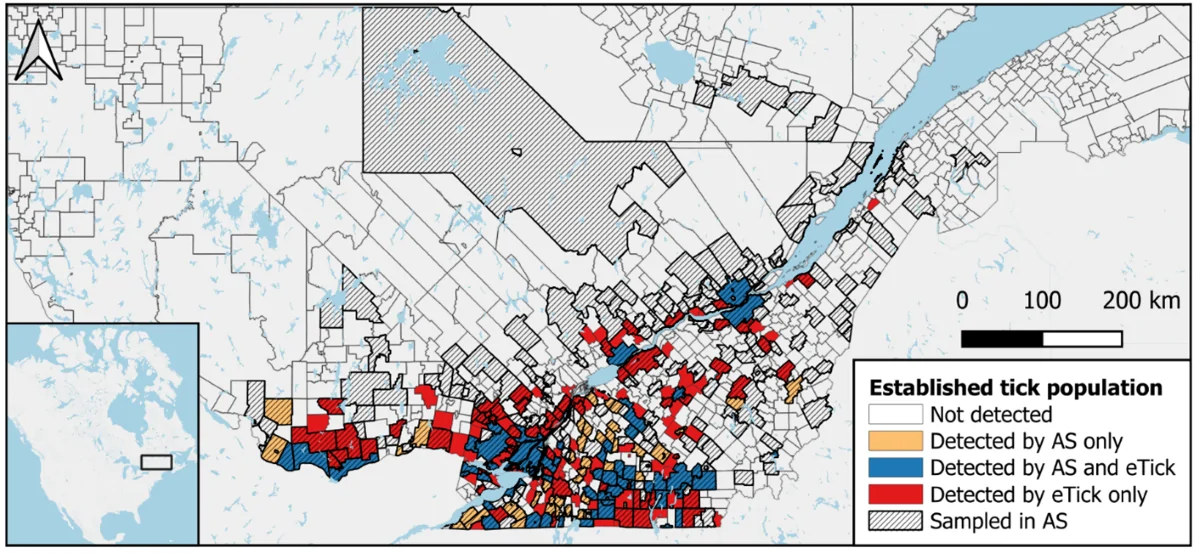
Comparison of established blacklegged tick (*Ixodes scapularis*) populations at the census subdivision (CSD) level in Quebec, Canada, between 2020 and 2023, based on active surveillance (AS) criteria (n=343 CSD sampled) and the eTick thresholds. Based on AS data, an *I scapularis* population is considered established if either 6 or more ticks have been found or at least two different life stages are found within the same year in a municipality. The eTick thresholds represent the number of submissions optimizing the balance between sensitivity and specificity and are derived from the top-performing model (C1) predicting established blacklegged tick populations using the eTick crowdsourced surveillance indicator, including all origins combined and socioecological factors. Thresholds are applied to CSDs according to climate suitability and population size, as detailed in Table 7. Depending on the CSD, thresholds range from 2 to 64 submissions to be considered indicative of an established population.

According to the active surveillance criteria for the presence of established blacklegged tick populations and the eTick indicator based on submission thresholds, all CSDs with established populations are located in the southern part of the province, up to the approximate 47˚N latitude of Quebec City (Figure 2). Among the 343 CSDs sampled through active surveillance, 248 (72.3%) were identified as positive for the presence of established tick populations by at least one of the 2 indicators. Of these 248 positive CSDs, 45 (18.1%) were identified by the active surveillance criterion only, 148 (59.7%) were identified by the eTick indicator only (using thresholds ranging from 2 to 64 submissions, as detailed in Table 7), and 55 (22.2%) were identified by both indicators (Figure 2).

## Discussion

### Principal Findings

This study demonstrates that crowdsourced eTick data can be used to develop robust, evidence-based indicators of blacklegged tick population establishment in Quebec. Although we did not detect spatial clusters in submission rates, we found that the rates were associated with socioecological factors, including climate suitability, population size, landscape configuration, and population socioeconomic characteristics. The results also highlighted key sources of variation that must be accounted for when deriving indicators from crowdsourced surveillance data. Building on these findings, we developed and tested indicators based on eTick submissions that were effective in detecting CSDs with established tick populations while accounting for confounding factors. Overall, the results support integration of crowdsourced surveillance data into the Canadian tick-borne disease surveillance framework as a complementary tool to traditional active surveillance. Previous studies using eTick data have applied different approaches to predict tick distribution or public health risk, providing useful points of comparison for the present findings. Bowser et al [89] assessed whether early eTick data (2018‐2020) could predict LD risk at the CSD level, in an endemic region of Quebec, Canada. They reported weaker associations and poorer model fit when eTick submissions were used as LD risk predictors compared to other indicators, attributing these results to the novelty of the platform at the time and variable levels of public adoption and participation. More recently, Westcott et al [90] used eTick submissions with other citizen science datasets to develop niche models predicting the current and future distribution of *I scapularis.* Both studies relied on raw submission data and did not explicitly account for socioecological factors influencing participation and reporting intensity, which may have limited risk signal detection in Bowser et al [89] and biased habitat suitability estimates in Westcott et al [90]. While these studies illustrate the potential utility of crowdsourced surveillance data, the lack of consideration of these factors may represent an important limitation. Indeed, results from this study indicate that eTick submission counts are influenced by both human and environmental factors, including population size, socioeconomic characteristics, and habitat suitability affecting tick occurrence and abundance. Submission rates likelihood reflect exposure risk among humans and companion animals as driven by behavior and tick presence and density, as well as factors influencing the decision to submit samples to eTick. Accordingly, the value of these data for contributing to surveillance programs depends on accounting for biases affecting submission rates.

Submissions to eTick were distributed across southwestern and central Quebec, corresponding to the known distribution range of *I scapularis* in the province [91]. Although no statistically significant spatial clusters were detected, there was geographic heterogeneity in submission rates for both submission types. Submission of ticks found attached to humans were mostly reported in the southernmost regions of the province, which are also the highest risk regions for LD (Outaouais, Montérégie, and Estrie [92]). In contrast, submissions of ticks found attached to animals were distributed across a wider geographic area, including regions located outside the currently defined endemic zones. Several of these regions include municipalities with lower risk levels for LD or are not considered endemic for LD according to the provincial risk classification [91]. Under this classification, municipalities are considered endemic when they meet one or more criteria regarding the number of declared human LD cases, the number of ticks collected through passive surveillance, or the number and stages of *Borrelia burgdorferi*–infected ticks collected in active surveillance [93]. Taken together, these patterns highlight the broader geographic scope of detections obtained through animal-origin submissions and suggest that combining submissions from both origins may provide complementary information for surveillance of tick presence beyond established endemic areas. As eTick data continue to accumulate over time, there may be sufficient statistical power to detect higher than expected submission rates, if present, which could help identify and test for currently unknown local factors (human and pet population, or tick) affecting exposure risk.

### Socioecological Factors Associated With Tick Submissions

In zero-inflated negative binomial models analyzing the factors associated with tick submissions, the total human population of CSDs was the strongest predictor of both submission likelihood and count, indicating that more populated areas are more likely to report tick presence. This result aligns with previous studies, which have shown that submission counts are generally proportionate to population size, where the odds of submission increase with the number of potential contributors [18-20,40,41]. These findings reinforce the importance of accounting for population size when analyzing passive surveillance data [18,19]. Additional socioecological factors also influenced submission occurrence and counts.

First, municipalities that accumulated more annual DD >0 °C were more likely to have tick detections. Ticks require ambient temperatures above 0 °C to quest for hosts, with warmer conditions accelerating interstadial development [94]. Globally warmer conditions throughout the year are thought to accelerate the completion of their life cycle and facilitate their establishment. Indeed, previous modeling and field studies have shown that a threshold of approximately 2800 DD >0 °C is needed for ticks to establish viable populations [67,95]. While DD >0 °C was a determinant of the presence of tick populations, it did not significantly affect submission counts once populations were established. This suggests that once tick populations are well established, other factors such as host densities, human-tick encounters, or participation behavior may drive the numbers of submissions to eTick.

The proportion of WUI in CSDs was positively associated with tick submission counts. This variable measures the degree to which human settlements (ie, neighborhoods where people live, infrastructures where people work, or roads providing access to the territory) are surrounded by natural areas, including woodlands, ecotones, and prairies, which may be suitable habitats for ticks and their hosts [65]. Results indicate that CSDs with higher WUI represent higher tick exposure risk areas [45]. Conversely, the WUI indicator was negatively associated with the occurrence of tick submissions (as denoted by a significant positive relationship with the likelihood of structural zeros), which is a surprising result that warrants further analysis. Here, we studied a region that may not be entirely suitable for tick establishment, with very different biomes across the territory [5]. It is unknown, for example, if boreal forest provides a habitat where *I scapularis* populations can become established [96]. Consequently, this result may reflect another underlying relationship not captured by our current indicators or may relate to the coarse ecological nature of the indicator, which is aggregated at the municipal level and includes all types of natural habitats. Moreover, ticks require specific ecological conditions regarding habitat type, host availability, and landscape connectivity at fine spatial scales, which likely could not be detected at the municipal scale used in this work [45,97,98].

Finally, CSDs with higher median household income and age were associated with more submissions. This aligns with other findings that citizen science initiatives tend to predominantly attract middle-aged and older groups and those with greater access to educational and financial resources [35,37]. This is a common point of critique [37,99] and an area where improvement is needed through initiatives aimed at reaching more diverse groups. Doing so would help ensure that the benefits of participation are more equitably distributed across the population [31] and that the indicators derived from these data are more representative of all areas and population profiles. Our proxy of general population awareness of ticks and LD, constructed using Google Trends data, showed a weak but positive relationship with submission counts in CSDs. This aligns with expectations that public awareness of the problematics under focus is generally associated with higher participation in citizen science initiatives [31,37,100]. However, several limitations inherent to Google Trends may have contributed to the weak association observed. Keyword-based indicators rely on specific search terms and may not capture the broader range of queries related to tick information seeking [101]. Additionally, Google search activity can be strongly influenced by short-term, media-driven attention, which may not reflect sustained awareness or a durable willingness to engage with surveillance initiatives such as eTick [101]. More broadly, public health intelligence derived from open-access, user-generated sources, such as Google Trends, is subject to participation bias, with contributions disproportionately originating from populations with greater internet access and digital literacy. As a result, these data may not accurately represent the population at risk to the public health threat [102,103]. Furthermore, Google Trends data can be unstable or imprecise when analyzed at small spatial scales or in areas with low population sizes, such as many CSDs, in the study area [104]. Collectively, these limitations may reduce the validity of this indicator as a proxy for awareness, as noted in previous studies [101]. Integrating complementary data from diverse sources, ranging from traditional public health surveillance systems to nontraditional data, such as open-access social media, may help strengthen signal detection and mitigate participation biases [102].

### The Detection of Established Blacklegged Tick Populations

To evaluate the ability to detect established *I scapularis* populations, we built and compared models using 1 of 3 crowdsourced surveillance indicators based on tick origin: ticks found attached to humans, animals, and from all origins combined. For each indicator, we compared simple models with more complex ones that accounted for socioecological factors associated with submissions in objective 1. In all cases, the complex models significantly outperformed simpler alternatives, including those accounting for population size or only using submission counts. While simple models can be useful for ease of interpretation and indicator development for public health use [18], the loss in performance here would be too high to justify this approach. This result highlights the relevance of analytically considering socioecological covariates when using passive or crowdsourced data, consistent with findings and approaches from other studies [20,41,44].

The 3 top-performing models yielded similar results: model A1, based on ticks found attached to humans and socioecological factors; model B1, based on ticks found on animals and socioecological factors; and model C1, based on all tick origins combined with socioecological factors. Given these similar performances and the complementarity observed in the geographic distributions of both submission types, we pursued subsequent analyses using the submissions from all origins to identify the threshold optimizing the detection of established tick populations. By doing so, we also ensured that all data collected by users were leveraged.

These analyses showcase a methodology for indicator development that can be adapted according to the objectives of the surveillance or research program and the epidemiological context. Previous Canadian passive surveillance studies have shown that ticks recovered from animals, particularly dogs, were effective at providing early signals and wider geographical extent of emerging tick populations [18-20,105]. This relationship has been attributed to differences in exposure, as dogs are generally more directly in contact with vegetation and leaf litter, where they are more likely to be bitten by questing ticks, even with low tick densities [13,14]. However, ticks recovered from animals may be less specific indicators than those from humans, as very low tick densities detected through animals may reflect adventitious ticks transported by migratory birds rather than established tick populations [18,19]. On the other hand, our analyses suggest that ticks from animals can be efficient at detecting emerging tick populations where active surveillance may not be possible or may be insufficient. Although active surveillance remains the most standardized method available for tick surveys, its efficacy depends on multiple factors, including vegetation type and weather conditions before and during sampling [106-108]. Drag sampling may be less effective for detecting emerging tick populations in northern Quebec, where coniferous forests and dense understory vegetation dominate, compared to deciduous forests in southern Quebec [106-109]. This highlights that both active and crowdsourced surveillance indicators may have strengths and limitations and that we would benefit from both by developing integrated indicators [15,100,110].

### Thresholds of Submissions Optimizing Tick Population Detection Accuracy

To identify submission thresholds that optimize tick population detection, we searched for cutpoints that maximized sensitivity, specificity, or both, using models based on submission types. In the best-performing model, a threshold of 3 submissions (rounded from 3.32) maximized both sensitivity and specificity for detecting tick populations. At this threshold, both sensitivity and specificity reached 76%. These levels are comparable to those of a previous study conducted in Quebec, which used passive surveillance to predict the presence of established *I scapularis* populations, as measured by active surveillance carried out from 2007 to 2008 [20]. That study identified an optimal threshold of 0.073 (Predicted Linear Index), which predicted the presence of ticks in the environment with 74.19% sensitivity and 73.68% specificity, using a similar methodology that maximized both indicators. Their model included passive surveillance data, an index of environmental suitability based on cumulative annual DD >0 °C, and an index estimating the number of ticks arriving on migratory birds. Overall, our results of crowdsourced surveillance shared similar characteristics and limitations with comparable traditional passive surveillance datasets from other Canadian studies: more adult female ticks were submitted than immature ticks, more ticks were recovered from animals than from humans, and similar factors affected the submission data [14,18-20]. The predictive performance of this dataset in identifying established tick populations, as confirmed by active surveillance, was consistent with that of traditional passive surveillance. Therefore, our analysis supports the validity of crowdsourced data as a complement to traditional active and passive surveillance. We believe these approaches should remain complementary, as each provides distinct information. Active surveillance provides standardized measures of tick density and confirmation of locally reproducing populations, while crowdsourced data offer broader geographic coverage and rapid detection of emerging areas [111], and traditional passive surveillance also more readily enables pathogen testing, which is generally not feasible with crowdsourced submissions. Future work should include an assessment of how these data should be used to build indicators that are adaptive to evolving epidemiological context, such as the one prevailing in Canada [11,105].

The optimal thresholds of submissions maximizing tick population detection accuracy were highly variable across the study area. This variability reflects the province’s pronounced heterogeneity regarding population size, land cover, climate, and socioeconomic characteristics. To facilitate interpretation, we exemplified what these thresholds would be across different groups of CSDs, regrouped by population size and climate suitability. Climatically suitable regions for ticks are concentrated in the southern parts of Quebec. These areas are well-known endemic zones for *I scapularis* and LD, including the highest incidence regions of Estrie and Montérégie [56]. Most CSDs in these regions have small population sizes and are suburban or rural. These areas also have a high proportion of WUI, meaning there is significant overlap between tick-suitable natural environments and human habitation. In such contexts, only a very small number of tick submissions was sufficient to indicate the likely presence of an established tick population. This suggests that even limited crowdsourced surveillance signals in these areas should raise awareness about possible reproducing tick populations and emerging risk of tick-borne disease [112]. In contrast, large population centers in climatically suitable areas required a higher threshold of submissions to balance sensitivity and specificity. In climatically unsuitable regions, a similar threshold was identified for small population centers, while the highest one was identified for medium population centers. These findings suggest that in emerging areas, a high number of ticks must be submitted before suspecting an established population, which may indicate that adventitious ticks are detected well before actual establishment occurs. Alternatively, it could reflect a mismatch between active and passive surveillance data in these regions. For example, active surveillance may fail to detect ticks in more northern environments due to limitations in sampling sensitivity, especially in the dense understory vegetation of coniferous forests [106,108], or because of lower sampling effort deployed in these areas. In contrast, passive surveillance may be less affected by these limitations, as it relies on ticks encountered by the public or professionals across a wider range of habitats and conditions.

This study has several limitations that should be considered when interpreting its findings. First, the spatial accuracy of eTick submissions implies an inherent level of uncertainty, as reported locations reflect the user’s presumed site of tick acquisition and cannot be assumed to be exact. These locations remain subject to recall and information bias, as users may have incomplete recollection of where exposure occurred or may infer locations based on routine activities. Spatial accuracy is expected to be higher when ticks are detected promptly after exposure or when individuals perform regular tick checks and are aware of their recent movements [28]. Excluding submissions from users reporting recent travel history mitigated the impact of this source of uncertainty; however, it was not possible to fully control for this limitation with the available data. Second, uncertainty in indicator precision may arise from spatial aggregation at the CSD level and from the use of active surveillance as an imperfect reference standard. Ecological processes relevant to tick establishment operate at finer spatial scales than those captured here, and active surveillance may fail to detect established populations under certain environmental or sampling conditions. Together, these limitations reflect the challenge of relying on relatively coarse spatial indicators. Likewise, although we attempted to account for participation biases to which crowdsourced initiatives are subject by including sociodemographic covariates in our modeling framework, residual bias likely remains. Finally, the generalizability of the proposed indicators beyond Quebec and over time will require further evaluation. Differences in surveillance systems, population engagement, ecological contexts, and public awareness across provinces or countries may affect indicator performance. Over time, the usefulness of a crowdsourced approach such as eTick depends on sustained participation from both the general public and professionals [113]. Since future engagement is unknown and may not remain stable over time, the continued usefulness of this surveillance method cannot be guaranteed. Regular reassessment will therefore be necessary to ensure data quality and long-term relevance.

### Conclusions

In this study, we analyzed the patterns in blacklegged tick submissions to the platform eTick in Quebec, Canada. This work introduces a rigorous analytical framework for interpreting crowdsourced surveillance data by comparing submission rates with long-term active surveillance. A novel contribution is using a socioecological approach to adjust for potential participation bias in eTick submission rates. Unlike prior Canadian tick passive surveillance studies, which rely primarily on traditional surveillance methods or that use unadjusted crowdsourced data, our approach explicitly incorporates socioecological factors associated with reporting into the modeling framework. This work demonstrates that crowdsourced surveillance data, when appropriately modeled, can yield reliable inferences about disease vector distributions. It provides a methodological approach applicable to other medically important vector species and geographic contexts, and contributes to improving the robustness of ecological inference of vector distributions. Specifically, our findings show that eTick can effectively complement traditional passive surveillance methods for detecting established blacklegged tick populations. Although this analysis focused on *I scapularis*, eTick allows the collection of all tick species and could therefore support monitoring of other medically important species in Canada, such as *Ixodes cookei, Dermacentor variabilis, Rhipicephalus sanguineus*, or *Amblyomma americanum*. These contributions have practical implications for public health practice. More confident identification of tick distributions supports targeted public health actions and improved surveillance strategies. The broad geographic coverage of eTick makes it well suited for early detection of emerging tick-borne diseases in undersurveyed regions. Integrating eTick data into formal surveillance systems could significantly enhance Canada’s capacity to detect those emerging risks in a cost-effective way. Future work should focus on expanding the analytical framework to other tick species and pathogens, conducting field validation of predicted tick populations using active surveillance, and improving the representativeness of reporting populations across diverse and historically excluded communities.

## Supplementary material

10.2196/90734 Multimedia Appendix 1 Ethics review exemption from Bishop's University Research Ethics Board.

## Abbreviations

AUC: area under the curve
CSD: census subdivision
DD: degree days
INSPQ: Institut National de Santé Publique du Québec
IRR: incidence risk ratio
LD: Lyme disease
OR: odds ratio
WUI: wildland-urban interface
