# Supplementary material for: Assessing the Effectiveness of Crowdsourced Data to Detect Established Tick Populations in Quebec, Canada: Retrospective Ecological Study
Source: JMIR Public Health Surveill. 2026 Aug 19;12:e90734. doi: 10.2196/90734 (PMC13489290; doi:10.2196/90734)
Supplement: Supplementary file 1 [file publichealth-v12-e90734-s001.pdf]

March 23st, 2023

Dr. Jade Savage
Professor, Biological Sciences
Bishop's University
2600 College Street
Sherbrooke, QC J1M 1Z7

SUBJECT: eTick Data publication

Dear Dr. Jade,

The Research Ethics Board (REB) has reviewed your application regarding the need for protocol approval prior to the publication of surveillance data collected through the eTick platform.

The REB unanimously decided that you did not need ethics approval for the non-public and public data in eTick.

If you have any questions about the REB's decision and process, please contact the Office of Research and Graduate Studies.

We wish you the best of luck with this very interesting project.

Sincerely,

Alex Miltsov, Ph.D. & Joachim Jean-Jules, Ph.D.
Chair, Research Ethics Board — Director Research Office and Graduate Studies
